# Indirect assessment of an interpretation bias in humans: neurophysiological and behavioral correlates

**DOI:** 10.3389/fnhum.2013.00272

**Published:** 2013-06-12

**Authors:** Anita Schick, Michèle Wessa, Barbara Vollmayr, Christine Kuehner, Philipp Kanske

**Affiliations:** ^1^Department of General Psychiatry, Section for Experimental Psychopathology and Neuroimaging, Center for Psychosocial Medicine, Heidelberg UniversityHeidelberg, Germany; ^2^Department of Clinical Psychology and Neuropsychology, Institute for Psychology, Johannes Gutenberg-University MainzMainz, Germany; ^3^Department of Psychiatry and Psychotherapy, Central Institute of Mental Health, Medical Faculty Mannheim/Heidelberg UniversityMannheim, Germany; ^4^Department of Social Neuroscience, Max Planck Institute for Human Cognitive and Brain SciencesLeipzig, Germany

**Keywords:** ERP, N200, LPP, cognitive bias, rumination, reflective pondering

## Abstract

Affective state can influence cognition leading to biased information processing, interpretation, attention, and memory. Such bias has been reported to be essential for the onset and maintenance of different psychopathologies, particularly affective disorders. However, empirical evidence has been very heterogeneous and little is known about the neurophysiological mechanisms underlying cognitive bias and its time-course. We therefore investigated the interpretation of ambiguous stimuli as indicators of biased information processing with an ambiguous cue-conditioning paradigm. In an acquisition phase, participants learned to discriminate two tones of different frequency, which acquired emotional and motivational value due to subsequent feedback (monetary gain or avoidance of monetary loss). In the test phase, three additional tones of intermediate frequencies were presented, whose interpretation as positive (approach of reward) or negative (avoidance of punishment), indicated by a button press, was used as an indicator of the bias. Twenty healthy volunteers participated in this paradigm while a 64-channel electroencephalogram was recorded. Participants also completed questionnaires assessing individual differences in depression and rumination. Overall, we found a small positive bias, which correlated negatively with reflective pondering, a type of rumination. As expected, reaction times were increased for intermediate tones. ERP amplitudes between 300 and 700 ms post-stimulus differed depending on the interpretation of the intermediate tones. A negative compared to a positive interpretation led to an amplitude increase over frontal electrodes. Our study provides evidence that in humans, as in animal research, the ambiguous cue-conditioning paradigm is a valid procedure for indirectly assessing ambiguous cue interpretation and a potential interpretation bias, which is sensitive to individual differences in affect-related traits.

## Introduction

Affective states, including depression, can strongly affect cognitive processes, such as attention, memory, appraisal, and decision-making (Mathews and Macleod, 1994; Beck, 2008; Gotlib and Joormann, 2010; Disner et al., 2011). It has been proposed that a negatively biased interpretation of ambiguous situations results from facilitated attentional processes through emotions (affective priming theories; Bower, 1981; Isen and Daubman, 1984; Isen et al., 1987). This theoretical consideration originates from the semantic network theory, which assumes that associated memories are more easily accessible through a process of “spreading activation” (Anderson and Bower, 1973). In that respect, cognitive theories of depression posit that negative schemata, which are dysfunctional mental representations about the self, trigger a mood congruent interpretation of a distinct situation as good or bad, which itself has consequences on the emotional state of an individual (Beck, 1976). An enduring vicious circle of negative interpretation bias and negative emotional states might then lead to the development of psychopathological conditions, such as affective disorders (Mathews and Macleod, 2005). Indeed, some empirical evidence for negative attention, memory, and interpretation bias related to depression has been provided; however, the results are mixed, probably due to specifics in the selection of stimulus material and assessment of the bias. While studies using questionnaires with ambiguous stories were able to detect a negative interpretation bias in depression (Butler and Mathews, 1983; Berna et al., 2011), other studies that used measures like response latency or startle reflex were only in part successful. Lawson and Macleod (1999) studied the naming latency of words in positive or negative valence presented after an affective prime sentence and found no relation to scores in the Beck Depression Inventory (BDI; Beck et al., 1996). In contrast, participants with a higher BDI score showed larger startle reflex amplitudes elicited by ambiguous merge words compared to neutral stimuli (Lawson et al., 2002). This is in line with the hypotheses of a negative interpretation bias in depression as the startle reflex amplitude is known to be increased after negative stimuli (Bradley et al., 1990; Lang et al., 1990).

Apart from clinical depression, individual coping style has been proposed to influence the interpretation of a situation as positive or negative. Lyubomirsky and Nolen-Hoeksema (1995) have shown that rumination, a coping style that refers to focusing one's attention and thoughts on negative aspects of a situation (Nolen-Hoeksema et al., 2008), leads to more negative interpretations of hypothetical situations. Using more explicit measures of cognitive bias, Kuehner and Huffziger (2012) showed that an induced ruminative self-focus after negative mood induction significantly increased dysfunctional depressiogenic attitudes in healthy individuals.

The heterogeneity of results in clinical as well as analogous samples (e.g., healthy individuals with elevated induced or naturally occurring negative mood), might, at least in part, result from methodological difficulties with experimental tasks that were used to assess biased information processing (see above). In the present study, we therefore adopted an ambiguous cue-conditioning paradigm from animal research that indirectly assesses biased information processing. In an acquisition phase, participants first learn to discriminate two tones of different frequency, which are followed by either a positive or a negative consequence. This part of the paradigm is similar to affective (or evaluative) conditioning which has been shown to be effective in various fields of research (De Houwer et al., 2001). Using a learning procedure similar to affective conditioning and pairing stimuli with reinforcers has repeatedly led to valence transfer as reported in the visual (Stolarova et al., 2006; Schacht et al., 2012) and auditory domain (Laufer and Paz, 2012). In a second phase of the paradigm participants are confronted with additional tones of intermediate frequency that are not reinforced. The response to these ambiguous tones is used as an indicator of an interpretation bias.

This experimental setup has several advantages. First, the auditory cues are indeed neutral in the beginning of the experimental procedure and have no negative or positive connotation. Also, as the intermediate tones are never followed by feedback, they are truly ambiguous which is essential for a cognitive bias to affect decision-making. This is in contrast to a study by Anderson et al. (2012), who applied a similar paradigm to assess emotional biases. In this study, however, the intermediate tones were also reinforced, which renders them non-ambiguous and, therefore, did not allow for the detection of an inherent interpretation bias. Second, this experimental setup was initially developed in rodents (e.g., Harding et al., 2004; Enkel et al., 2010). Its adaptation to human research paves the way for translational research that offers new possibilities for identifying neural and molecular mechanisms underlying biased information processing as well as the potential of developing new treatment strategies. Using such an ambiguous cue-conditioning paradigm, Enkel et al. (2010) successfully distinguished between congenitally non-helpless and helpless rats, which served as an animal model of depression. Moreover, Richter et al. (2012) showed that the negative bias of helpless rats was decreased after enrichment supporting the idea of using such bias as a measurement sensitive to depression treatment.

To also elucidate the neural time-course underlying biased information processing, we assessed event-related brain potentials (ERPs) of the EEG. Promising potentials include the N2 component, peaking around 200 ms post-stimulus over fronto-central electrode sites, which is associated with cognitive control and response conflict (Folstein and Van Petten, 2008). In the present study, ambiguous stimuli make demands on cognitive control processes (e.g., in cancelling a prepared response) and induce response conflict due to perceptual similarity and unclear response demands. N2 amplitude increases have been reported for increasing perceptual similarity (Folstein and Van Petten, 2004) and for increasing difficulty to discriminate ambiguous stimuli (Szmalec et al., 2008).

In addition, a positive deflection of the ERP starting around 300 ms post-stimulus has been consistently related to emotion and arousal (see Olofsson et al., 2008). As discussed by Kissler et al. (2009), this potential has been variously termed P3, late positive potential (LPP), or late positive complex (LPC). For the present study, we will use the term LPP for this positivity. There is evidence showing it to be increased for emotional stimuli (Foti et al., 2009; Hajcak et al., 2010; Kaestner and Polich, 2011) even when controlling for arousal (e.g., Rozenkrants and Polich, 2008; Kaestner and Polich, 2011; Feng et al., 2012) and it is also related to subjective intensity ratings of emotion (Cuthbert et al., 2000). Interestingly, it has also been reported to differentiate between negatively and positively conditioned stimuli (Schacht et al., 2012).

Late positive ERP components with a maximum over frontal electrode sites have also been associated with executive processes involved in categorization (Folstein and Van Petten, 2011) and there is evidence for an interaction between categorization and emotional valence modulating the LPP. In categorization tasks, negative stimuli have been found to elicit larger LPPs than either positive or neutral stimuli (Kanske and Kotz, 2007). Here again, the interpretation of the ambiguous tones may be reflected in the LPP amplitude. Therefore, in the present study, the LPP may be increased for reference tones because of their association with reward and punishment and could also reflect the differential processing of positively and negatively interpreted ambiguous tones.

In sum, the main goal of the present study was to test the described ambiguous cue-conditioning paradigm in humans. Therefore, we aimed at (1) establishing that the intermediate tones are perceived as ambiguous by comparing reference and intermediate tones, and (2) elucidating the processing of negatively and positively interpreted ambiguous stimuli. As pointed out above, interpretation of ambiguous stimuli is influenced by affective states and cognitive styles. We therefore assessed current affect, depression, and rumination. We hypothesized that ambiguity of the intermediate tones would be reflected in uncertain response choices, increased response times, and increased amplitudes of the N2 due to difficult discriminability and unclear response demands resulting in response conflict. We also expected LPP amplitudes to be increased for the non-ambiguous reference tones because of their greater behavioral relevance and associated affective salience. We further hypothesized the specific interpretation of ambiguous stimuli to be reflected in differential ERP responses, specifically LPP amplitudes, which might show increases for negatively interpreted tones.

## Materials and methods

### Participants

Participants were recruited via advertisements at the universities of Mannheim and Heidelberg. They received course credits and obtained the monetary gain from the ambiguous cue-conditioning task according to their task performance (see below for details). In total, 20 participants (10 women) with a mean age of 24.2 years (*SD* = 9.1) took part in the experiment. All had normal or corrected to normal vision and normal hearing. One participant reported to be left-handed. Since we had no lateralization hypotheses and as the results did not change, when excluding this participant, we report data with this participant included. None of the participants reported a history of head injuries, tinnitus, or mental disorders. After being informed about the experiment the participants gave written informed consent. The study was approved by the local Ethics Committee of Heidelberg University and was conducted in accordance with the Declaration of Helsinki.

### Materials

**Figure 1:**
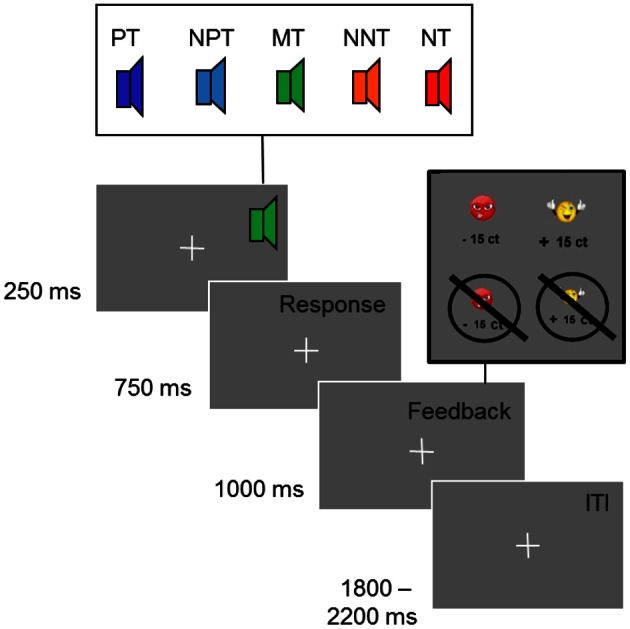
**Ambiguous cue-conditioning paradigm.** PT, positive tone; NPT, near-positive tone; MT, middle tone; NNT, near-negative tone; NT, negative tone. Participants were instructed to press a button after each tone to obtain reward or avoid loss of reward (0.15 €). After the button press participants received a feedback. In case of a correct identification of the positive tone, they saw a smiley indicating a monetary gain. For a wrong button press or no response, they saw a crossed smiley indicating that they had missed the chance to earn money. A correct identification of the negative tone was followed by a picture of a crossed frowney indicating that they had successfully avoided loosing money; for a wrong button press, participants lost money and saw a frowney. No feedback was presented after intermediate tones (NPT, MT, NNT) and after 4% of the reference tones (PT, NT). The inter-trial-interval (ITI) was jittered between 1800 and 2200 ms.

Stimuli consisted of five sinusoidal tones with a fundamental frequency between 1000 and 1164 Hz. They were selected so that all tones had a distance of 0.25 Bark (*f*_1_ = 1000 Hz, *f*_2_ = 1038 Hz, *f*_3_ = 1078 Hz, *f*_4_ = 1120 Hz, *f*_5_ = 1164 Hz). The total duration of the tones was 250 ms with a linear ramp of 20 ms. For feedback a yellow smiley or a red frowney were presented (see Figure 1).

### Experimental procedure

Participants were tested in an electrically shielded room in a single experimental session. They were seated in front of a monitor screen (1 m distance). To adjust the loudness of the tones to the individual hearing level, participants were presented a sinusoidal tone of 1000 Hz, which decreased in loudness, and pressed a button as long as they heard the tone. This procedure was repeated 10 times. The intensity of the test tones was then scaled according to the individual hearing level (Moore, 2003). The experimental task was to discriminate two reference tones (tone 1 and 5) by pressing one of two buttons with their right index or middle finger, respectively. One of the reference tones is referred to as “positive tone” (PT) as it acquired positive valence over the course of the experiment through positive feedback (smiley) and monetary gain (15 cents) after a correct button press. If participants responded incorrectly to this tone, they were informed that they had “missed the chance to win” money. In this case, a picture of a crossed smiley was shown. The other reference tone is referred to as “negative tone” (NT), as participants lost 15 cents when they pressed the incorrect button and negative feedback (frowney) was presented. By pressing the correct button to the NT, participants could prevent money loss and were presented with a crossed frowney and the information that loss of money had been avoided. If participants did not press any button within a response window of 1 s, they either lost money when the NT was presented or missed the chance to win money when the PT was presented. Each trial was comprised of a tone lasting 250 ms, a response window of 750 ms, the following feedback lasting 1 s and, finally, a jittered inter-trial interval of 2 s on average (randomly selected between 1800 and 2200 ms) (see Figure 1). Participants were randomly assigned to one of four counterbalanced conditions with respect to the finger used for button presses and the fundamental frequency of PT and NT.

During a brief learning and a training session, participants learned to discriminate PT and NTs. First, both tones were presented five times each and participants were told how to respond (learning session). Second, discrimination of reference tones was practiced with 40 randomized trials (training session). In the experimental test phase, three additional tones were presented (66 times each) in addition to the two reference tones (PT, NT; 282 times each). The three additional tones were intermediate in frequency (see section Materials) and labeled near-positive tone (NPT), middle tone (MT), and near-negative tone (NNT). The three intermediate tones were not followed by any feedback to render them fully ambiguous. All tones were presented in pseudo-randomized order. Furthermore, during the test phase 24 (4%) of the reference tones (12 PT, 12 NT) were also presented without feedback to cover the presence of intermediate tones. Thus, a total of 222 tones were presented without feedback, another 540 trials (270 PT, 270 NT) were presented with positive or negative feedback. All tones without feedback were less frequent than reference tones with feedback to cover their presence and to keep the participants motivated. Participants were instructed to respond to each tone by pressing one of the two buttons and they were informed that not every trial would have a feedback. The test phase was divided into six blocks of 127 trials, each lasting about 8 min. At the end of each block participants had a break of 2 min in which they were informed about the total amount of money won up to that point.

### Questionnaires

Several questionnaires were used to explore inter-individual differences in emotional state and trait variables. We measured current depression with the German version of the Beck Depression Inventory II (Beck et al., 1996; Hautzinger et al., 2006), a 21 item self-report questionnaire. To investigate strategies for coping with depressive symptoms participants completed a German version of the Response Style Questionnaire (RSQ; Nolen-Hoeksema, 1991), which tests for two subcomponents of rumination: “reflective pondering” and “brooding” (10 items; Gonzalez et al., 2003; Kuehner and Huffziger, 2012). Furthermore, participants completed the Positive and Negative Affect Scale (20 Items; Watson et al., 1988) immediately before the ambiguous cue-conditioning task.

### EEG recording

During the ambiguous cue-conditioning task, a continuous 64 channel EEG was recorded using Ag/AgCl-electrodes positioned according to the international 10/20 system. The signals were amplified by Neuroscan Synamp amplifiers (Compumedics, Charlotte, NC, USA), digitized at a rate of 500 Hz and recorded by Neuroscan Scan 4 Acquire software (Compumedics, Charlotte, NC, USA). The right mastoid was used as on-line reference and an electrode positioned on the sternum was used as ground electrode. Another electrode was placed on the left mastoid (for offline re-referencing). Horizontal eye movements were recorded from two electrodes placed lateral to both eyes, while two electrodes placed above and below the right eye registered vertical eye movements. Impedances of all electrodes were kept below 15 kOhm.

### Data analysis

For the EEG data analyses, Brain Vision Analyzer software (Brain Products GmbH, Munich) was used. The pre-processing of the EEG data included re-referencing to the mean of the mastoids and down-sampling to 200 Hz. Then, the data were filtered (0.1–30 Hz) to remove high- and low-frequency waves and the data were visually inspected to check for artifacts. To correct for eye movement artifacts, we performed an independent component analysis (Comon, 1994). In a next step, segments of 1200 ms starting 200 ms pre-stimulus and ending 1000 ms after stimulus onset were created. Using the semiautomatic artifact rejection tool, segments were excluded if the minimum and maximum amplitude in a segment differed by more than 300 μV. To obtain event-related potentials (ERPs), the segments were averaged relative to a 200 ms pre-stimulus baseline.

For the statistical analyses of behavioral, questionnaire, and ERP data, SPSS Statistics 18 (SPSS Inc., Chicago, IL, USA) was used. To test for effects of ambiguity we compared behavioral and ERP responses to the reference and to the intermediate tones. Because of the very low number of incorrect responses to the reference tones, only the correct response trials were included for analyses of reaction time and ERP data. To analyze the participants' response choice, a difference score between the frequencies of the two response options (positive, negative) was calculated, reflecting the degree of uncertainty in associating a tone with a response. This difference score was then compared between reference and intermediate tones.

To test for effects of interpretation biases, we analyzed the responses to the three intermediate tones since the participants' response reflects the categorization of the ambiguous tones as either predicting reward or punishment. Here, we calculated 3 × 2 repeated measures ANOVAs with the factors tone (NNT, MT, NPT) and response (positive, negative). Also, to obtain an overall measure of the cognitive bias, which can be correlated with questionnaire scores, we calculated a bias score defined as the mean of all responses to the three intermediate tones. A response to avoid punishment (negative response) was calculated as −1 while a response to obtain reward (positive response) counted +1. A positive bias score indicates more positive than negative responses, while a negative bias score indicates more negative than positive responses to the ambiguous tones. An independent sample *t*-test was computed to test for gender differences in the bias score. To test if the bias changed during the test phase, a One-Way ANOVA with the factor block (1–6) was calculated.

In this study, ERP analyses focused on N2 and LPP. Based on the literature (Folstein and Van Petten, 2008) we extracted the mean activity in the time window from 180 to 240 ms post-stimulus for analyzing the conflict-related N2 component. For LPP analyses, we first calculated an omnibus ANOVA of the mean activity with the factors tone (NNT, NPT, MT), response (positive, negative), and electrode for consecutive time windows of 100 ms up to 1000 ms. These analyses showed a significant response by electrode interaction in the time window from 300 to 700 ms. For the analyses of the ambiguity effect we chose a shorter time window from 0 to 500 ms for the omnibus ANOVA with the factor ambiguity (reference tones, intermediate tones) and electrode since analyses of the later time windows would be confounded by feedback-related activity that occurred on average 540 ms post-stimulus (as a feedback was only presented after reference tones, not after the intermediate tones). Based on the results obtained here we focused further analyses on the time window from 300 to 500 ms. We then exported mean activity in the time range 300–500 ms (early LPP) and 300–700 ms (late LPP) and performed analyses per electrode. Based on these analyses we defined two regions of interest (frontal: F1, Fz, F2, FC1, FCz, and FC2; posterior: P1, Pz, P2, PO3, POz, and PO4) that we included in further analyses.

To link behavioral data with ERP results and questionnaire data, we computed bivariate Spearman correlations. For all analyses significant thresholds of *p* < 0.05 were used and significant main effects and interactions were followed up with Bonferroni corrected *post-hoc* paired comparisons or contrasts. A Greenhouse-Geisser correction was applied when necessary.

## Results

### Behavioral findings

#### Response choice

**Figure 2:**
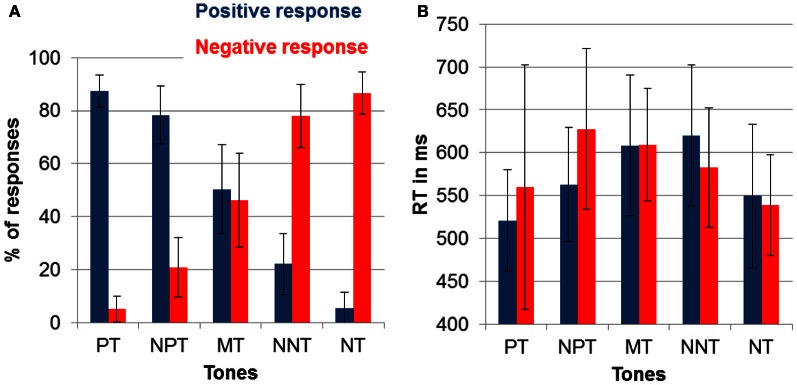
**(A)** Percentage of chosen responses (and SD) to avoid punishment (negative response) and obtain reward (positive response) for each of the five tones. **(B)** Mean reaction times (and SD) to the five tones.

Participants were well able to discriminate the two reference tones as indicated by 86.9% (*SD* = 20) correct responses in the training session. In the following test phase, the percentage of correct responses to the reference tones was similarly high (mean percentage of correct responses: 87.0%; *SD* = 7), despite the presentation of additional intermediate tones (see Figure 2A).

To test for the effects of ambiguity on response choice, we compared responses to the reference and to the intermediate tones. Specifically, we compared the absolute difference between the percentage of positive and negative button presses. For the reference tones, this yielded a mean difference score of 81.73% (*SD* = 11.56). For the intermediate tones the index was 45.68% (*SD* = 13.70), indicating a more undetermined response pattern. A repeated measures ANOVA with the factor ambiguity (reference tones, intermediate tones) was significant [*F*_(1)_ = 143.73; *p* < 0.001; partial η^2^ = 0.88].

To check for effects of interpretation biases, we compared the number of negative and positive responses to the three intermediate tones. A repeated measures ANOVA with tones (NNT, MT, NPT) and responses (negative, positive) yielded a significant effect of tone [*F*_(1.07)_ = 12.13; *p* < 0.01; partial η^2^ = 0.39], which points to differences between NNT and MT (*p* < 0.001), as well as NPT and MT (*p* < 0.001) as indicated by pairwise comparisons. A significant tone by response interaction [*F*_(1)_ = 189.72; *p* < 0.001; partial η^2^ = 0.91] was driven by a higher percentage of positive responses to NPT and a higher percentage of negative responses to NNT [*F*_(1)_ = 355.40; *p* < 0.001; partial η^2^ = 0.95].

#### Reaction time

Figure 2B displays the reaction time data for all tone and response combinations. To test for the effect of ambiguity on reaction times, we again compared reference and intermediate tones. This effect was significant indicating that participants responded faster to the reference compared to the intermediate tones [*F*_(1)_ = 27.64; *p* < 0.001; partial η^2^ = 0.59].

To test for the effect of interpretation biases, the three intermediate tones were compared with repeated measures ANOVA with the factors tone (NPT, MT, NNT) and response (positive, negative). This analysis showed a significant tone by response interaction [*F*_(2)_ = 18.45; *p* < 0.001; partial η^2^ = 0.49]. *Post-hoc* contrasts showed that this interaction was due to faster responses to obtain reward than to avoid punishment after NPT [*F*_(1)_ = 19.44; *p* < 0.001; partial η^2^ = 0.51] and faster responses to avoid punishment than to obtain reward after NNT [*F*_(1)_ = 11.85; *p* < 0.005; partial η^2^ = 0.38]. Positive and negative responses to MT were equally fast (*p* > 0.90).

#### Individual differences in bias score

**Figure 3:**
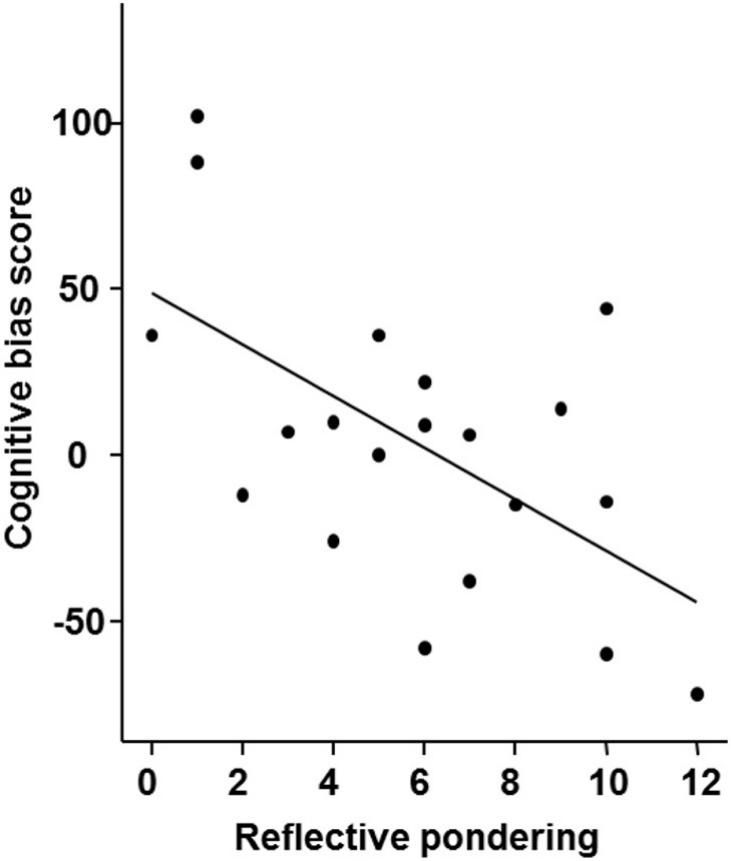
**Correlation of the cognitive bias score with the reflective pondering subscale of the Response Style Questionnaire (*p* < 0.05; ρ = −0.501)**.

In the current sample the bias score was slightly positive with a mean of 3.95 (*SD* = 44.8) but not significantly different from 0 [*t*_(19)_ = 3.94; *p* = 0.70]. To test if the bias changed throughout the experiment, we calculated a One-Way ANOVA with the factor block, which was not significant indicating constant interpretation of the intermediate tones across the six experimental blocks (*p* > 0.5). We also observed no gender differences (*p* > 0.5). Furthermore, there was no significant correlation of cognitive bias with current mood (PANAS) and depression (BDI; all *p* > 0.5). We did, however, observe a significant correlation between the bias score and the reflective pondering subscale of the RSQ, indicating that participants with a higher score in reflective pondering displayed a more negative bias (ρ = −0.50; *p* = 0.025; see Figure 3) while the brooding subscale did not correlate with the bias score (*p* > 0.5).

### ERP results

Across conditions the following ERP components were detected: a negative deflection peaking around 200 ms after tone onset (N2) and a positive deflection starting around 300 ms after tone onset (LPP).

In order to define the latency range of these components we calculated several omnibus ANOVAs per electrode. Besides the effect for electrode in each time window, we obtained a significant effect for ambiguity from 400 to 500 ms [*F*_(60)_ = 10.24; *p* < 0.01; partial η^2^ = 0.39] and significant interactions for ambiguity and electrode [300–400 ms: *F*_(60)_ = 1.83; *p* < 0.001; partial η^2^ = 0.1; 400–500 ms: *F*_(60)_ = 2.56; *p* < 0.001; partial η^2^ = 0.14]. Further analyses focused on the time window from 300 to 500 ms.

For the interpretation bias effect omnibus ANOVAs revealed main effects for electrode in each time window and in addition effects for response in the time window from 300 to 400 ms [*F*_(1)_ = 4.4; *p* < 0.05; partial η^2^ = 0.22] and from 600 to 700 ms [*F*_(1)_ = 11.76; *p* < 0.01; partial η^2^ = 0.4]. Further, the analyses showed a significant response by electrode interaction in the time windows from 300 to 400 ms [*F*_(60)_ = 2.6; *p* < 0.001; partial η^2^ = 0.14], from 400 to 500 ms [*F*_(60)_ = 2.0; *p* < 0.001; partial η^2^ = 0.11], from 500 to 600 ms [*F*_(60)_ = 1.9; *p* < 0.001; partial η^2^ = 0.11] and from 600 to 700 ms [*F*_(60)_ = 1.68; *p* < 0.001; partial η^2^ = 0.1]. Thus, analyses focused on the time window from 300 to 700 ms.

#### Ambiguity effect

**Figure 4:**
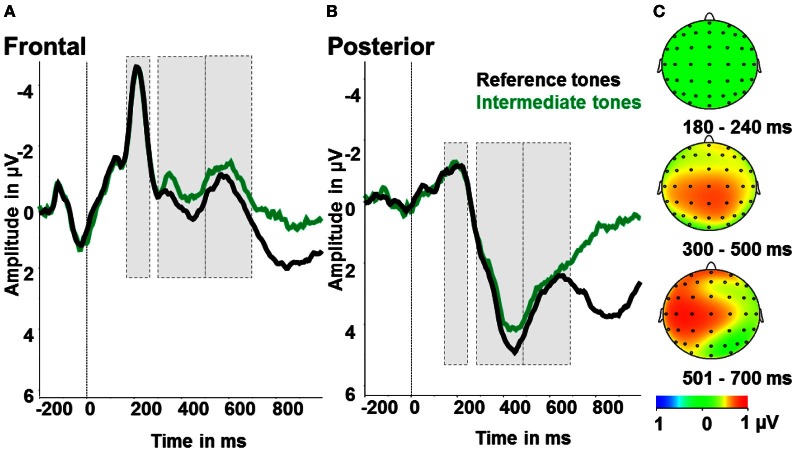
**Ambiguity effect: ERPs after reference tones (black) and intermediate tones (green). (A)** Event-related activity averaged over frontal electrodes (F1, Fz, F2, FC1, FCz, FC2). **(B)** Event-related activity averaged over posterior electrodes (P1, Pz, P2, PO3, POz, PO4). **(C)** Maps display the activity difference of the reference tones and correct responses minus ambiguous tones and all responses in μV in the time windows 180–240 ms (N2), 300–500 ms (early LPP) and 501–700 ms (late LPP) post-stimulus.

To test for the effects of ambiguity, we calculated an ANOVA with the factors ambiguity (reference tones, intermediate tones) and region (anterior, posterior). For the early LPP time window (300–500 ms), we identified significant main effects of ambiguity [*F*_(1)_ = 6.0; *p* < 0.05; partial η^2^ = 0.27] and region [*F*_(1)_ = 54.75; *p* < 0.001; partial η^2^ = 0.78]. As shown in Figure 4, early LPP amplitudes were larger for reference compared to ambiguous tones and over posterior compared to anterior electrodes. The interaction of ambiguity and region was not significant (*p* > 0.1). For the N2, only a significant effect of region [*F*_(1)_ = 79.45; *p* < 0.001; partial η^2^ = 0.82] with larger N2 amplitudes over frontal compared to posterior electrodes was found. Further main effects or interactions were not significant (all *p* > 0.5, see Figure 4).

#### Interpretation bias effect

**Figure 5:**
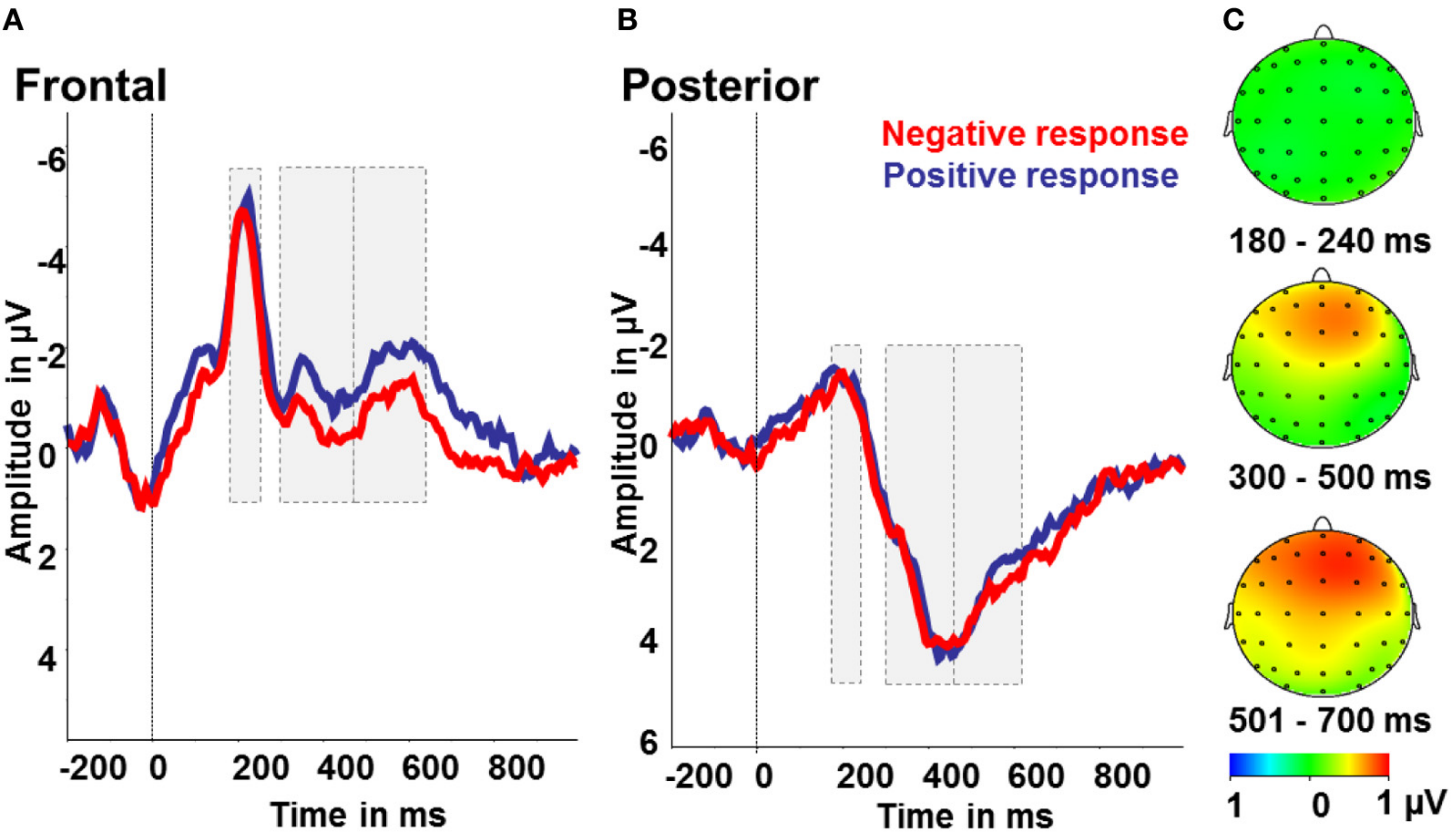
**Interpretation bias effect: ERP amplitudes for positive (blue) and negative responses (red) to the intermediate tones. (A)** Frontal region of interest. **(B)** Posterior region of interest. **(C)** Activity difference of ambiguous tones and positive responses minus ambiguous tones and negative responses in μV in the time windows 180–240 ms (N2), 300–500 ms (early LPP) and 501–700 ms (late LPP) post-stimulus onset.

To test for indicators of different processing of positively or negatively interpreted stimuli, we compared intermediate tones with positive and negative responses. Therefore, we conducted repeated measures ANOVAs with the factors tone (NPT, MT, NNT), response (positive, negative), and region (frontal, posterior). For the LPP in the time window 300–700 ms post-stimulus there were significant effects of response [*F*_(1)_ = 4.55; *p* < 0.05; partial η^2^ = 0.22] with larger amplitudes after negative responses and a main effect of region with larger amplitudes over posterior electrode sides [*F*_(1)_ = 65.08; *p* < 0.001; partial η^2^ = 0.80]. Besides, there was a significant response by region interaction [*F*_(1)_ = 11.21; *p* < 0.01; partial η^2^ = 0.41]. Over frontal electrodes, amplitudes were increased after negatively, as opposed to positively, categorized intermediate tones [*F*_(1)_ = 11.11; *p* < 0.01; partial η^2^ = 0.41], while there were no effects over posterior electrode sites (all *p* > 0.5; see Figure 5). For the N2, a significant effect of region [*F*_(1)_ = 63.29; *p* < 0.001; partial η^2^ = 0.78] with larger N2 amplitudes over frontal compared to posterior electrodes was found. Further main effects or interactions were not significant in this time range (all *p* > 0.5, see Figure 5).

## Discussion

The current study employed an ambiguous cue-conditioning paradigm for the indirect assessment of an affect-related interpretation bias and investigated the related neurophysiological correlates with EEG. In contrast to instrumental conditioning procedures, this paradigm comprised a second stage introducing additional stimuli intermediate in frequency to the learned ones. Ambiguity of these intermediate tones could be established with participants responding slower and with less certainty when confronted with the intermediate tones. In the current sample of healthy individuals, a small positive cognitive bias was observed which was associated with inter-individual differences in ruminative coping style, i.e., reflective pondering. Higher scores in reflective pondering were related to a more negative bias. Also, the data yield insight into the time-course of ambiguous stimulus interpretation showing decreases in LPP amplitudes after ambiguous tone presentation, but no N2 effect. Moreover, we observed differences in ERP amplitudes depending on the interpretation of the ambiguous stimuli: frontal LPP amplitudes were increased for negatively compared to positively interpreted intermediate tones.

### Ambiguity effect

For the validity of the present paradigm it is essential that the intermediate tones are perceived as ambiguous with regard to what potential outcome they predict. Evidence for this is the increased response uncertainty that participants showed by selecting positive and negative responses equally often after the intermediate tones, while the responses to the reference tones were either clearly positive or negative. Additionally, response times were longer for intermediate tones also indicating increased response uncertainty (Szmalec et al., 2008; Anderson et al., 2012).

The collected ERP data can shed light on the time-course of processing ambiguity in the intermediate tones. In contrast to our hypotheses, we observed no effect of ambiguity on the N2. As ambiguity has been conceptualized as representing a form of cognitive conflict (Szmalec et al., 2008), we would have expected to see increased N2 amplitudes for ambiguous vs. reference tones, analogous to incongruent vs. congruent stimuli in conflict tasks like the flanker or Stroop (van Veen and Carter, 2002). A critical difference from previous reports of N2 increases for ambiguous stimuli (Szmalec et al., 2008) is the affective context in the present study. Szmalec et al. (2008) also had participants differentiate two tones of variably perceptual similarity, but responses were not associated with reward or punishment. Positive and negative emotional stimuli, however, have been shown to modulate processing of cognitive conflict and the related N2 amplitude (Kanske and Kotz, 2010, 2011). In particular, the N2 is enlarged for stimuli of greater emotionality, reflecting increased recruitment of cognitive control processes (for an overview see Kanske, 2012). In the present study, it could be argued that the reference tones possess more emotional salience due to their association with potential monetary gain or loss, thus recruiting more cognitive control resources. This may have raised N2 amplitudes to the level of the ambiguous tones. The pattern of LPP amplitude changes corroborates this explanation. We observed increased LPP amplitudes for the reference compared to the intermediate tones, which suggests that the reference tones were perceived as more salient. The LPP has been consistently found to be increased for emotional and arousing stimuli of different modalities (Cuthbert et al., 2000; Schupp et al., 2003; Foti et al., 2009; Schacht and Sommer, 2009; Hajcak et al., 2010). In addition, P3 which peaks in a similar time range has been associated with task relevance (for a review see Kok, 2001). In the present study, task relevance is arguably higher for the reference tones, as they are followed by monetary gains and losses, while the responses to the intermediate tones are without consequences.

In sum, the ERP data suggest that the reference tones in the present task were of higher salience than the intermediate tones, reflected in increased LPP amplitudes, which may have overridden an ambiguity effect in the N2 time window.

Since participants were presented with a visual feedback after the reference tones (which occurred on average 540 ms after stimulus onset), but not after the intermediate tones, the ERP cannot be meaningfully interpreted in the late LPP time window. The late positive deflection which is increased for reference compared to intermediate tones from 540 ms post-stimulus onwards is most likely due to this visual stimulation.

### Interpretation bias effect

A second question we addressed concerned the differences in processing between positive and negative interpretations of the ambiguous intermediate tones. The absence of a strong overall bias means that about half of the intermediate tones were interpreted negatively and positively. This pattern varied, however, as NPT and NNTs were interpreted more often as positive and negative, respectively. Interpretations in the opposite direction (e.g., a negative response to a NPT) were also slowed down. The major question here was whether the decision to respond to a tone positively or negatively is associated with differential processing of the tones. The effect of tone interpretation on LPP amplitudes suggests that this is the case. The amplitudes were increased for tones that were subsequently responded to with a negative compared to a positive button press. This direction of the effect falls in line with several previous studies that showed enlarged positivities for different types of emotional stimuli (Kanske and Kotz, 2007; Rozenkrants and Polich, 2008; Kaestner and Polich, 2011; Feng et al., 2012). The present data, however, add to this evidence that the top-down interpretation of the affective value of a certain stimulus yields similar brain responses as when the affective value is inherent in the stimulus. Previously, Schacht et al. (2012) found increased LPP amplitudes for stimuli with learned positive valence. The authors suggest that this finding might be due to better learning for the positive compared to negative reinforcers. The present results show an effect on LPP amplitude due to the interpretation and association of the intermediate tones with a certain valence. The fact that we find enlarged LPP amplitudes for negatively interpreted tones might be explained by task differences. In our study, participants received feedback on their response and thus learned a tone—response association leading to one positive and one NT. In contrast, Schacht et al. (2012) used picture sets of different valence (as rated a priori) and participants had to classify the pictures in positive, neutral, or negative without feedback. Beyond that, the focus of our analyses was on intermediate tones that were not reinforced in the acquisition phase. Here, we find processing differences apparent already from around 300 ms post-stimulus in the LPP. Even though the more anterior distribution of this component is not typical, some variability in the topography of valence effects in the P3 time window has been reported (Rozenkrants and Polich, 2008; Feng et al., 2012). Principal components analyses of valence-related ERP effects corroborate this, showing a number of late positivities that might only partially share neural generators because of different scalp distribution (Foti et al., 2009). The exact role of these differentiable components still needs to be specified, however.

### Interpretation bias and its relationship to affect-related variables

We suggested that valence is ascribed to the intermediate tones on the basis of an individual interpretation preference that biases cognitive processing. However, here we observed no significant correlations between current positive or negative mood or depression and interpretation bias, although this has previously been reported (Eysenck et al., 1991; Mogg et al., 2006; Anderson et al., 2012). The lack of a relationship between current mood and depressive symptoms with the interpretation bias in the present study might result from a very limited variance in these affect-related variables in young healthy individuals (e.g., BDI ranging from 0 to 8 on a scale with a maximum score of 63, see Table 1). Nevertheless, we did observe a significant negative correlation between the bias score and reflective pondering, a subcomponent of rumination. This might indicate that individuals with a stronger ruminative coping style show a more negative bias and vice versa. Joormann et al. (2006) have also studied the relation between cognitive bias and rumination. Here, an attentional bias toward sad faces correlated significantly with brooding, a second subcomponent of rumination as measured with the RSQ, but not with reflective pondering. From this finding, the authors concluded that there might be functional as well as dysfunctional components of rumination. However, in depressed patients both rumination subscales (brooding and reflective pondering) were increased compared to healthy controls (Joormann et al., 2006). There are several explanations for the finding of a relationship between reflective pondering and a negative interpretation bias, while no such relationship was found between brooding and biased information processing. First, questionnaire data show that the variance for brooding was much smaller than for reflective pondering, limiting the potential to find a correlation. Second, whereas in clinical depression reflective pondering might represent the more adaptive ruminative coping style (in comparison to brooding), it still indicates a ruminative coping style that is maladaptive when compared to more adaptive cognitive coping strategies, such as positive reappraisal, positive refocusing, or focusing on planning. Our result of a negative correlation between reflective pondering and the interpretation bias is in line with previous studies relating cognitive bias and rumination (Gotlib and Joormann, 2010; Koster et al., 2011) and encourages further research with clinical samples using the described paradigm as it suggests that a maladaptive, depressive cognitive style is related to negative interpretation bias.

**Table 1:** **Questionnaire data**.

	**Minimum**	**Maximum**	**Mean**	***SD***
BDI	0	8	2.85	2.46
RSQ_R	0	12	5.80	3.40
RSQ_B	1	8	4.45	2.46
PA	18	39	28.65	6.72
NA	10	18	11.00	1.89

*Range, mean, and standard deviation (SD) of participants' scores in the Beck Depression Inventory (BDI), Reflective pondering subscale (RSQ_R), and Brooding subscale (RSQ_B) of the Response Style Questionnaire, and positive (PA) and negative affect (NA) assessed before the measurement with the Positive and Negative Affect Scale (PANAS)*.

That, on a group level, we did not observe a significant interpretation bias may be plausible, given the fact that we investigated a group of healthy individuals that rather tend to show a positive bias (Deldin et al., 2001). Further, it is supposed that cognitive biases result from depressiogenic schemata and that they are not active until triggered by a negative event or a negative mood state (Scher et al., 2005). Thus, negative mood or thought induction may be necessary to elicit a negative cognitive bias in control participants. With the induction of self-focused thoughts which are similar to ruminative thinking, Hertel and El-Messidi (2006) observed more negative interpretations of ambiguous homographs in dysphoric students. Future research could combine mood induction procedures with the present paradigm to test for changes in the measured bias.

## Limitations

Although the present study provides a validation of an animal experimental setup that allows the indirect assessment of an interpretation bias and gives new insights into the time-course of ambiguous cue processing, a number of limitations have to be pointed out. First, we did not assess other, more explicit measures of cognitive bias in addition to the ambiguous cue-conditioning task, which could have added some external validity to the present results. Second, we did not collect valence rating for the tones after the conditioning paradigm, which could have corroborated their acquired valence status. In a later yet unpublished study we included valence ratings. In this study participants ascribed more positive valence to the PT than to the NT and the intermediate tones. The NT did not differ in valence which might be due to the fact that only false responses to the NT had negative consequences. A direct loss after the NT would be a stronger negative feedback and more comparable to the punishing effect of an electric shock in the study by Enkel et al. (2010). Apart from the valence transfer to the intermediate tones their categorization might also be influenced by the sensory resemblance of the NPT to the PT and the NNT to the NT. Sensory similarity might facilitate the affective interpretation of these tones or affective interpretation might partly be a consequence of the sensory similarity. If sensory similarity was the only basis for decision-making then the responses would be identical to the ones after the corresponding reference tones. The present results indicate that responses to these tones are biased by both the frequency information of the tones and top-down interpretations. In case of the MT sensory resemblance plays no role since these tones resemble neither the PT nor the NT. Responses to these tones might therefore underlie a cognitive bias more strongly. In addition, the intermediate tones might differ in their degree of ambiguity. Although the lack of feedback after all three intermediate tones leads to uncertainty as seen by an increase in reaction time, the sensory resemblance of NPT and NNT might facilitate response selection. Thus, MT represents the highest level of ambiguity. In the present study the number of MT was too small for statistical analyses but further studies could increase the number by only presenting MT and no NNT or NPT. Another limitation of the paradigm might be that it lacks a neutral condition. Presenting another tone which is either followed by neutral feedback or where the participant does not need to respond would further corroborate the affective conditioning procedure. Finally, as the present study was designed to validate the employed experimental task and to delineate the neurophysiological mechanisms of ambiguous cue processing and biased interpretation of ambiguous cues, we were not able to detect a relation of the interpretation bias with depression measures. As this was probably due to the small variance in depression scores in the present sample, future studies should test clinical samples with the procedure. Although the correlational findings of the present study suggest an association between interpretation bias and rumination, our sample size was very small. Besides, we did not correct for multiple comparisons underlining the rather exploratory nature of our findings although it is under debate if Bonferroni corrections are appropriate (Perneger, 1998). To corroborate our findings mood or rumination inductions (e.g., Huffziger and Kuehner, 2009) would be necessary. But, we also have to point out that the literature on cognitive biases in depression is inconsistent (for reviews see Dalgleish and Watts, 1990; Gotlib and Joormann, 2010). Especially studies using implicit measures of cognitive bias fail to detect a negative interpretation bias (Lawson and Macleod, 1999) even after negative mood induction (Bisson and Sears, 2007).

## Conclusion

The present study aimed at establishing an ambiguous cue-conditioning paradigm in humans. Such an approach has the advantage that it assesses the interpretation bias indirectly, which yields it unaffected by demand effects or a priori connotations of the applied stimulus material (as is the case, for example, in words; Lawson and Macleod, 1999; or homophones; Mogg et al., 2006). Furthermore, it offers the possibility of testing for positive and negative biases by assigning affective significance (positive and negative, respectively) to two initially neutral tones through classical conditioning. After such an acquisition phase, the test phase introduced tones of intermediate frequency that served as a measure of interpretation bias since the response to these tones indicated the participants' expectation of a rewarding or potentially punishing event.

The results of the present study provide evidence that ambiguous cue processing and resulting interpretation bias is assessable by using the proposed ambiguous cue-conditioning task that has previously been established in animals. On a behavioral level, ambiguous stimuli led to uncertainty in their response options and longer reaction times. On a neurophysiological level, we observed no N2 differences, but increased LPP amplitudes for reference stimuli compared to ambiguous stimuli, suggesting greater task-relevance and emotional salience for the reward- and punishment-related stimuli. Interpretation of the ambiguous stimuli had an effect on LPP over frontal electrodes with increased amplitudes for a negative compared to a positive interpretation. This indicates early and prolonged differences in the activation of top-down interpretation mechanisms.

### Conflict of interest statement

The authors declare that the research was conducted in the absence of any commercial or financial relationships that could be construed as a potential conflict of interest.
